# Numerical and Experimental Investigation of Time-Domain-Reflectometry-Based Sensors for Foreign Object Detection in Wireless Power Transfer Systems

**DOI:** 10.3390/s23239425

**Published:** 2023-11-26

**Authors:** Martin Helwig, Yun Xu, Uwe Hentschel, Anja Winkler, Niels Modler

**Affiliations:** Institut für Leichtbau und Kunststofftechnik, Technische Universität Dresden, 01307 Dresden, Germany; yun.xu@tu-dresden.de (Y.X.); uwe.hentschel@tu-dresden.de (U.H.); anja.winkler@tu-dresden.de (A.W.); niels.modler@tu-dresden.de (N.M.)

**Keywords:** electric time domain reflectometry, wireless power transfer, wireless charging, inductive power transfer, foreign object detection, metal object detection

## Abstract

Foreign object detection (FOD) is considered a key method for detecting objects in the air gap of a wireless charging system that could pose a risk due to strong inductive heating. This paper describes a novel method for the detection of metallic objects utilizing the principle of electric time domain reflectometry. Through an analytical, numerical and experimental investigation, two key parameters for the design of transmission lines are identified and investigated with respect to the specific constraints of inductive power transfer. For this purpose, a transient electromagnetic simulation model is established to obtain and compare the sensor impedance and reflection coefficients with experimental data. The measurement setup is based on parametrically designed sensors in laboratory scale, using an EUR 2 coin as an exemplary test object. Consequently, the proposed simulation model has been successfully validated in this study, providing a comprehensive quantitative and qualitative analysis of the major transmission line design parameters for such applications.

## 1. Introduction

Foreign objects are considered a major concern in the safe application of a wireless power transfer system (WPTS). In particular, objects made from electrically or magnetically conductive materials interact strongly with the alternating magnetic when located on the ground assembly (GA). Due to the resulting eddy current and hysteresis losses, objects might heat up strongly. This is a safety hazard as those objects may damage the GA or even cause fire. Moreover, hot objects can lead to burns when touched. In order to mitigate these issues, technical solutions are required to either prevent or limit the exposition of foreign objects to strong magnetic field strengths.

One general approach is the prevention of hazardous strong magnetic field strengths per design; for instance, by either reducing the transfer power or reducing the air gap. An alternative solution is to utilize a monitoring device that can detect foreign objects in the vicinity of strong magnetic field interaction prior to or during power transfer [1].

In the literature, many foreign object detection (FOD) methods are known, which are divided into living object detection (LOD) and metal object detection (MOD) [2,3,4]. Based on their detection principle, such systems can be categorized into the three groups: system-parameter-based detection, wave-based detection and field-based detection.

System-parameter-based FOD monitors the effect of foreign objects on the electrical, thermal or mechanical parameters of the WPTS. Electrical parameters are usually current [5,6], power loss or efficiency [7], quality factor [8], coil inductance, frequency or phase [9]. Non-electrical parameters are, for example, temperature [10,11], weight or pressure.

Wave-based detection methods rely on imaging or thermal cameras [12,13,14] and ultrasonic [15] or radar sensors [16], and thus require the use of additional sensing equipment.

Field-based detection methods rely directly on the local interaction between the metallic object and the magnetic field. Such monitoring devices are usually realized as passive inductor loops, which are located below the surface area of the GA coil. Any electrically or magnetically conductive material (i.e., materials with a sufficient deviation in magnetic permeability or electric conductivity relative to air) changes the local magnetic field distribution of the GA coil or of a separate excitation coil, which can be detected as a change in induction voltage of a local sensor loop. Several monitoring device designs based on the field-based detection method have been proposed in the past [17,18,19,20,21,22,23,24,25]. However, these designs require multiple sensor coils to cover the entire surface area of the GA, which need to be interconnected in pairs to cancel out both the GA induction voltage as well as the external noise and have to be multiplexed to a detection circuit. Furthermore, significant effort is expended on blind spot mitigation [19,20], as neighboring coils also cancel each other out when an object is located precisely in between them and the induction voltage nullifies [17,23,25]. Moreover, such devices are in principle only capable of metal object detection.

In response to these limitations, this study introduces a new FOD technique for a WPTS that utilizes time domain reflectometry (TDR). This approach differs from current field-based detection systems by inherently providing a straightforward, non-overlapping sensor layout without blind spots, while it is able to correlate temporal resolution with spatial resolution to determine object locations. As an active method, it does not depend on an external GA magnetic field, allowing for object detection before initiating power transfer. Additionally, this method is capable of identifying and differentiating between metallic and non-metallic objects due to its fundamental operating mechanism.

To the best of the authors’ knowledge, no prior research has been carried out in the field of TDR-based object detection in connection with wireless power transfer (WPT) applications. A partially related study was carried out by Dominauskas et al. [26], employing a “snake”-curved sensor configuration to measure the distributed resin flow during a liquid composite molding process. Another related contribution was made by Kostka et al. [27], wherein TDR was utilized in order to identify touch events during human–machine interactions in robotics. TDR has also found extensive application in moisture sensors. Suchorab et al. [28] presented a surface sensor designed to measure the volumetric water content in concrete samples. A comprehensive review of TDR-based moisture sensing applications in porous media is presented by He et al. [29].

Given the absence of comparable work, the primary aim of this study is to establish a fundamental understanding of the sensor principle and identify specific key design parameters for FOD applications. This is achieved by conducting parametric studies of small-scale laboratory sensor designs, which are introduced in Section 2. Section 3 describes the analytical and numerical methods used for time-dependent impedance calculation, which serve as a reference for measurement data. The experimental setup is then explained in Section 4. Section 5 presents, compares and discusses the simulation and measurement results, followed by a conclusion in Section 6.

## 2. Proposed TDR Sensor Design

The measurement method of electrical TDR, which is well established in electrical engineering, enables the spatially resolved measurement of the electrical properties of a transmission line (TL) based on propagation times and reflection characteristics of the electrical signals fed in at the beginning of the line. By modifying at least one of the components of the TL, which serves as the sensing element, a physical quantity can be coupled to the electrical properties of the line (resistive coating, inductance coating, leakage coating and capacitance coating).

In the TDR method, a high-frequency signal (pulse signal) is injected into a TL. Reflections occur at inhomogeneities of the TL, which can be detected at the beginning of the line and displayed in a reflectogram in the time domain. Reflections occur as a result of discontinuities in the characteristic impedance along the TL. The impedance at the point of discontinuity differs from the characteristic impedance of the TL.

In principle, a sensor based on a TL is only capable of sensing changes in impedance along its one-dimensional path. Therefore, in order to create a FOD sensor that can be applied on a two-dimensional surface, the TL track must be arranged in the shape of an area-filling curve. Several area-filling curves, such as spiral curves, “snake” curves and fractal curves like the Hilbert curve, have been described in the literature. These curves map the (*x*,*y*) coordinates of a two-dimensional surface onto a one-dimensional coordinate along the line.

**Figure 1:**
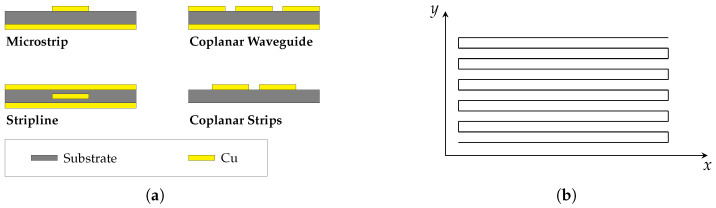
(**a**) Examples of commonly used printed circuit board (PCB)-based transmission line designs with and without a conductive ground plane in a cross-sectional view. (**b**) Area-filling, “snake”-curved path design of the sensor transmission line.

TLs are commonly used in various practical configurations, such as microstrips, coplanar waveguides, strip lines and coplanar strips (see Figure 1a). However, many of these configurations are unsuitable for the application of FOD sensors under the influence of strong external alternating magnetic fields. For example, coplanar waveguides typically rely on a conductive ground plane covering the entire back face of the substrate. This is disadvantageous for a couple of reasons. Firstly, such a conductive plane would shield the magnetic field of the GA, thereby preventing power transfer. Additionally, induced in-plane eddy currents would lead to a strong heat-up of the conductive sheet. Thus, transmission lines with a continuous ground plane cannot be utilized for this purpose.

In order to detect foreign objects, the TLs’ impedance has to change under the influence of the foreign object. In theory, the TL impedance is a function of three material properties: conductivity σ, relative permeability $\mu_{r}$ and relative permittivity $\epsilon_{r}$. However, for a change in impedance to occur, the electric and magnetic field components of the pulse wave must interact with the foreign object. As a result, the electromagnetic field must not be confined within the substrate, where an interaction with the foreign object is impossible.

However, an “open” designed transmission line is susceptible to electromagnetic interference, primarily to induced voltages, which disturb or even damage the measurement equipment. This needs to be minimized by optimizing and adapting the transmission line and curve design to the specific ground assembly. However, such optimizations or other means of interference suppression are not in the scope of this work and will be addressed in future research.

Unlike most known PCB-based transmission line designs, coplanar strips satisfy these requirements without the need for a solid, conductive ground plane. This is because coplanar strips do not require a conductive ground plane that covers the whole ground assembly, which would shield the magnetic field of the wireless charger and induce inplane eddy currents. Among area-filling curves, the “snake” curve (see Figure 1b) is a favorable choice as it can be easily implemented, parameterized and optimized to minimize the induction voltage caused by the magnetic field of the GA. Therefore, in this paper, the “snake” curve is utilized for the FOD sensor design.

Note that, in this study, all experiments are conducted without the presence of a magnetic field generated by the GA coil in order to perform measurements in a low-noise environment, serving as a reference for simulation models. Implementing the sensor principle discussed in real-world scenarios would require efficient noise reduction techniques, which are not covered in this study.

**Figure 2:**
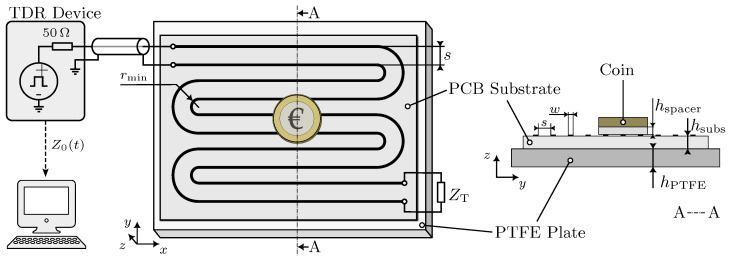
Schematic visualization of the FOD system setup in perspective view (**left**) and in cross-sectional view along the cutting line A (**right**).

The schematic representation of the complete system configuration is presented in Figure 2. The FOD-sensor is connected with a TDR device from Sympuls [30] through a coaxial cable of 50 Ω characteristic impedance. The TDR device generates rectangular pulses at a frequency of 24.4 kHz and a rise time (10 to 90%) of 80 ps. The sensor is terminated with a 100 Ω resistor, designated as $Z_{T}$, which is closer to the expected TL impedances, while also serving as a reference impedance different from the 50 Ω. In order to minimize reflections, the TL is bent with a minimum defined radius $r_{\min}$ of three times the copper strand width *w* [31]. The sensor is positioned on top of a plate of Polytetrafluoroethylene (PTFE) with $h_{\mathrm{PTFE}}=7\;mm$ thickness, which serves as a low-loss dielectric material with a well-defined dielectric constant. The PCB substrate of the sensor is made from RO4350B [32], which has a thickness of $h_{\mathrm{subs}}=0.3\;mm$ with a copper thickness of 35 μm. It has been chosen because the material properties are well known, it has low dielectric losses and it is easily available through manufacturers. In this work, an EUR 2 coin is utilized as a foreign object for all experiments. It is separated from the sensor surface using a variable thickness PTFE spacer in the shape of the coin. The experimentally measured, time-dependent wave impedance $Z_{0}\left(t\right)$ is finally transferred to a measurement computer via USB.

Following an initial parameter study of the prototype, two critical parameters that affect the sensitivity of the FOD were identified: the spacing between the two traces and the distance between the detection object and the transmission line. These parameters form the foundation for the parametric investigation presented in this study. The corresponding values for this parametric study were selected within an arbitrary range, derived from the coin size, and are provided in Table 1.

## 3. Analytical and Numerical Studies

This section describes the analytical and numerical methods used to obtain the wave impedance $Z_{0}$ of the TL. These methods rely on electromagnetic properties of the used materials, such as the relative electric permittivity $\epsilon_{r}$, the relative magnetic permeability $\mu_{r}$ and the electric conductivity σ. The values for these parameters are provided in Table 2 and serve as the foundation for all the methods used. Note that parameters for conductive materials are not provided in this table because they are neither required for the analytical nor the numerical methods, as can be seen in the subsequent sections. In total, an analytical calculation as well as a two-dimensional and a three-dimensional finite element method (FEM) simulation are conducted. The purpose of the analytic and two-dimensional methods is to determine a reference impedance for the coplanar strips configuration, which is then used to validate the accuracy of the three-dimensional simulation model. In the three-dimensional simulation, the sensor is simulated in the time domain, with and without the presence of foreign objects, to obtain a spatially and temporally resolved impedance. All simulations are conducted in the FEM simulation software COMSOL Multiphysics v6.0.

### 3.1. Analytical Calculation of Transmission Line Impedance

An analytical solution for the wave impedance of coplanar strips is well known in the literature [34] and can be calculated analytically following Equation (1):

(1) $$\begin{array}{cc} Z_{0} & =\frac{120\pi }{\sqrt{\varepsilon_{\mathrm{eff}}}}\frac{K(k)}{K(k^{\prime })}, \end{array}$$

where

$$\begin{array}{c} k=\frac{s}{b}\;\;\;\;\mathrm{and}\;\;\;\;k^{\prime }=\sqrt{1.0-k^{2}}. \end{array}$$

**Figure 3:**
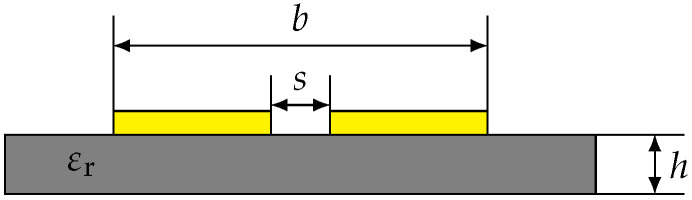
Cross-sectional schematic of coplanar strips on a substrate with a relative permeability of $\varepsilon_{r}$.

Here, K is the elliptic integral of the first kind, the variable *s* denotes the distance between the copper strands and *b* is the total width of the transmission line, as shown in Figure 3. The effective permittivity, denoted by $\varepsilon_{\mathrm{eff}}$, incorporates the geometric features of the surrounding materials like air as well as a PCB substrate of the thickness *h* via Equation (2) as follows:

(2) $$\begin{array}{c} \varepsilon_{\mathrm{eff}}=1+\frac{\varepsilon_{r}-1}{2}\frac{K\left(k^{\prime }\right)K\left(k_{1}\right)}{K\left(k\right)K\left(k_{1}^{\prime }\right)}, \end{array}$$

where

$$\begin{array}{c} k_{1}^{\prime }=\sqrt{1.0-k_{1}^{2}}\;\;\;\;\mathrm{and}\;\;\;\;k_{1}=\frac{\sinh(\frac{\pi s}{4h})}{\sinh(\frac{\pi b}{4h})}. \end{array}$$

### 3.2. Two-Dimensional Calculation of Transmission Line Impedance

The wave impedance of a coplanar strip can be determined using the telegrapher’s equations, yielding the Equation 3, which relies on the distributed parameters of inductance (*L*), capacitance (*C*), resistance (*R*) and conductance (*G*). These parameters can be obtained through electrostatic and magnetic simulations conducted within a two-dimensional domain. The angular frequency in Equation (3) is denoted as ω, whereas the imaginary unit is denoted as *j*.

(3) $$\begin{array}{c} Z_{0}=\sqrt{\frac{R+j\omega L}{G+j\omega C}} \end{array}$$

**Figure 4:**
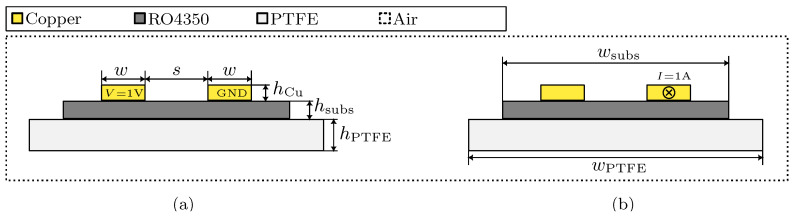
Cross-sectional schematic view of (**a**) the 2D electrostatic simulation model and (**b**) the 2D magnetic simulation in frequency domain.

This work assumes that all dielectric materials exhibit ideal behavior, where neither polarization losses nor conductance losses are accounted for. As a result, the conductance parameter *G* is zero. The capacity *C* is derived from an electrostatic simulation according to Figure 4a, where a voltage difference of 1 V is applied between the two conductors. The simulation is governed by Equation (4), where E is the electric field strength, Φ the electric potential, $\rho_{V}$ the volume charge density and ϵ the absolute electric permittivity.

(4) $$\begin{array}{c} \nabla \;\cdot \;E=\frac{\rho_{v}}{\epsilon },\;\;\;\;\;\;E=-\nabla \Phi \end{array}$$

The parameters *R* and *L* are derived from a magnetic simulation in the frequency domain according to Figure 4b following Equation (5), where H is the magnetic field strength, B the magnetic flux density, A the magnetic vector potential and J the current density, to which $J_{e}$ contributes as external current density. The external current density is set by a 1 A external current flowing through the cross section of the right conductor.

(5) $$\begin{array}{c} \nabla \times H=J,\;\;\;\;\;\;\;\;B=\nabla \times A,\;\;\;\;\;\;\;\;J=\left(\sigma +j\omega \epsilon \right)E+J_{e},\;\;\;\;\;\;\;\;E=-j\omega A \end{array}$$

In both simulations, an air domain is modeled around the transmission line and the substrates. 

### 3.3. Three-Dimensional Simulation of TDR-Based FOD

**Figure 5:**
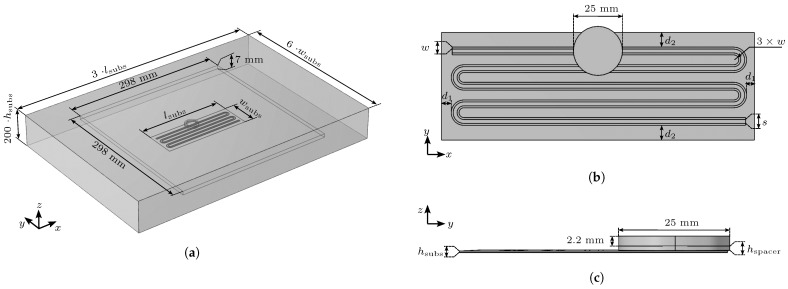
Geometry and dimensions of the model. (**a**) Perspective overview of the geometry. (**b**) Detailed top-down view and (**c**) side view of the sensor PCB with dimensions.

The 3D simulation model of the FOD sensor consists of five main parts: the air domain, the PTFE plate, the detection object (coin), the PTFE spacer and the sensor, as shown in Figure 5. The sensor for FOD is positioned at the center of a square PTFE plate with a side length of 298 mm each and a thickness of 7 mm. The sensor and the PTFE plate are enclosed by an air domain. The sensor consists of a substrate plate and two parallel meander copper traces. The distance between the sensor surface and coin is set by PTFE spacers of defined thicknesses. Although an EUR 2 coin is composed of two different alloys, in the model, it is only represented as a single cylindrical domain with a perfect electric boundary condition. The diameter of the coin is 25.75 mm and the thickness is 2.2 mm, as shown in Figure 5b,c.

The size of the air domain, the substrate and the position of the coplanar strips on the substrate will change due to the varying trace widths or the varying spacing between them. The size of the air domain is set to at least include the PTFE plate and the sensor. All sensors’ copper traces have a defined width of w=1 mm and are centered with respect to the substrate, which is indicated by the distances $d_{1}$ and $d_{2}$ in Figure 5b. The spacings from s=2 mm to s=10 mm are designated as s2 to s10. The geometric parameters corresponding to the above requirements for s2 to s10 are listed in Table 3.

For the computational part of TDR-based FOD in the three-dimensional case, mainly the vector potential formulation for transient electromagnetic waves is applied, following Equation (6):

(6) $$\nabla \times \left(\frac{1}{\mu_{r}}\left(\nabla \times A\right)\right)+\mu_{0}\sigma \frac{\partial A}{\partial t}+\mu_{0}\epsilon_{0}\epsilon_{r}\frac{\partial^{2}A}{\partial t^{2}}=0$$

**Figure 6:**
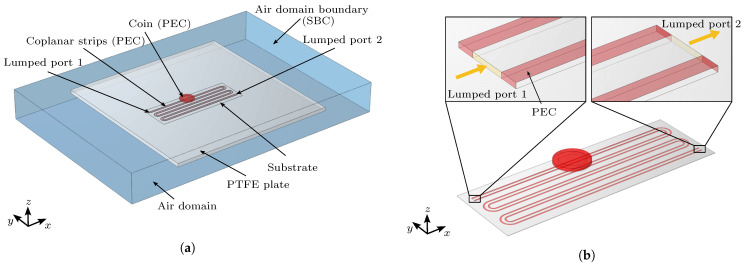
Domain and boundary definitions of the model. (**a**) Perspective overview of the geometry. (**b**) Detailed perspective view of the sensor PCB. For better recognition, the PEC boundary conditions has been colored red and the lumped port boundary conditions has been colored yellow.

The signal is input and terminated by the lumped port surface boundary conditions in COMSOL, which are depicted in Figure 6b as yellow rectangular surfaces bridging the two traces of the coplanar strip line. At the lumped port boundary condition, the characteristic impedance *Z* is defined as:

(7) $$Z=\frac{V}{I},$$

where *V* denotes the voltage at the port and *I* is the current. In the simulation, the lumped port 1 is designated as the input port, while the lumped port 2 is specified as the terminal port. A step function is used as an input signal to excite the coplanar strip line. The voltage amplitude of the step function is set to 1 V and the rise time is set to 0.2 ns. These values are obtained from the manual of the TDR measuring device [30]. The impedance of lumped port 1 is set to 50 Ω and the impedance of lumped port 2 is set to 100 Ω, according to the experimental setup.

In the simulation of electromagnetic waves, the perfect electric conductor (PEC) boundary condition is applied to all metallic parts (depicted as red areas in Figure 6), including the microstrip line and the coin in the three-dimensional simulation model. The PEC boundary condition is set as:

(8) n×E=0

where n is the unit normal vector of the boundary surface and E is the electric field. The sensor and PTFE plate are surrounded by the air domain. Electromagnetic waves propagate in the air domain and pass through the air domain boundary without reflection. The exterior surfaces of the air domain are set as the scattering boundary condition (SBC), which is an absorbing boundary used to describe an open space. The calculation of the SBC is defined as:

(9) $$\mu_{0}n\times H+\frac{\mu_{0}}{Z_{c}}n\times \left(E\times n\right)-\frac{\sigma Z_{c}}{2\mu_{r}}n\times \left(A\times n\right)=0$$

(10) $$Z_{c}=\sqrt{\frac{\mu_{0}\mu_{r}}{\epsilon_{0}\epsilon_{r}}}$$

Electromagnetic waves in coplanar strips propagate via a quasi-transverse electromagnetic (quasi-TEM) mode. Since the speed of electromagnetic wave propagation in the substrate differs from that in the air, it is necessary to calculate the phase velocity of the electromagnetic wave in the substrate to obtain the traveling time. The effective dielectric constant required for the computation of the phase velocity of coplanar strips is derived using Equation (2). The phase velocity is then calculated using the expression provided in [34]:

(11) $$v_{p}=\frac{c}{\sqrt{\epsilon_{\mathrm{eff}}}},$$

where $v_{p}$ is the phase velocity in the substrate and *c* is the speed of light. The maximum simulation time is approximated from the traveling time of the wave from lumped port 1 to lumped port 2 and then back to lumped port 1. However, in order to obtain complete simulation results, the approximated maximum simulation time is defined to be at least $12\;\cdot \;l_{\mathrm{subs}}/v_{p}$, which comfortably accounts for twice the TL length plus the corners.

**Figure 7:**
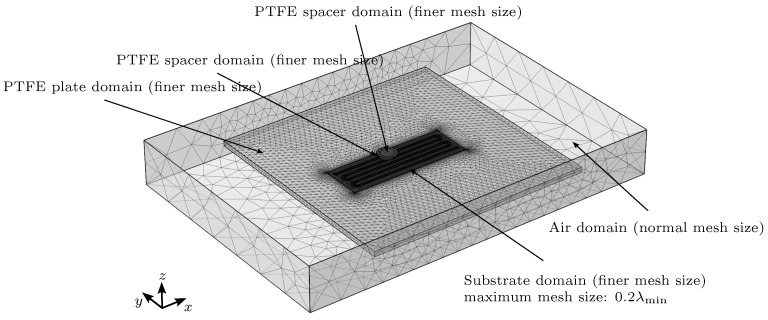
Meshing of the 3D simulation model.

When solving electromagnetic wave problems using the finite element method (FEM), a mesh that is too large can lead to an incorrect resolution of the waves in the signal. Conversely, a mesh that is too small can lead to a longer simulation time, improving the accuracy of the results. The rise time of the signal is 0.2 ns, which leads to a corresponding maximum signal frequency of 5 GHz, designated as $f_{\max}$. Accordingly, the minimum wavelength in the dielectric substrate is set to $\lambda_{\min}=v_{p}$/$f_{\max}$. Following a parameter study of the mesh size, the maximum mesh element size in the dielectric substrate, designated by $e_{\max}$, is set to 0.2$\;\cdot \;\lambda_{\min}$. In the simulation, all domains are meshed with COMSOL’s “Finer” mesh size setting, except the air domain, where the "Normal" mesh size setting is applied (as shown in Figure 7).

Similar to defining the maximum mesh size in space in solving electromagnetic wave problems, it is also important to define a suitable time step. A too small time step would unnecessarily lead to a longer simulation time, while a too large time step would lead to inaccurate solutions. The maximum time step chosen for the simulation is set to 0.2$\;\cdot \;e_{\max}/v_{p}$ and has been determined to yield the best results in the simulation.

## 4. Experimental Setup

**Figure 8:**
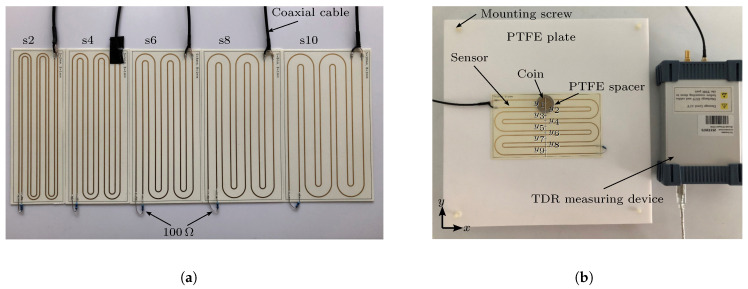
(**a**) Overview of the manufactured parametrically designed sensor samples. (**b**) Overview of the experimental setup.

As mentioned in the previous sections, two major parameters (spacing *s* and the distance between object and transmission line $h_{\mathrm{spacer}}$) were determined, which have an impact on the sensitivity of the FOD. The prototypes in the “snake” curvature have been produced on a laboratory scale, and comply with the parameters given in Table 1. Figure 8a shows the prototypes with the trace width of 1 mm and different spacings.

The prototype for the laboratory experiment consists of three main components: the sensor, an external 100 Ω termination resistor and a 50 Ω coaxial cable. The sensor PCBs are manufactured by Multi Leiterplatten GmbH (Brunntal, Germany) and the substrate material is Rogers 4350, obtained from Rogers Corporation (Chandler, AZ, USA). The FOD experiment device consists of 5 main parts: the PTFE plate, the detection object (coin), the sensor, the PTFE spacer and a TDR measuring device D-TDR 3000 from Sympuls [30].

For the experiments, the coin is placed in nine different locations on the sensor, as shown in Figure 8b. The impedance of each position and three different distances (0.5 mm, 1 mm and 2 mm) between the coin and the sensor are measured. These distances are realized with the coin-shaped PTFE spacers of defined thickness, which also prevent direct electric contact between the coin and the sensor. The purpose of the plastic screws in the PTFE plate is to maintain an air gap between the plate and the table. By doing so, these screws minimize the unknown dielectric influence of the surrounding environment on the impedance measurement results, thus creating a well-defined and specified environment for the purpose of easier simulation validation.

During the measurement, the coaxial cable of the prototype is connected to the TDR measuring device, which, on the other end, is connected to the PC via USB. The corresponding TDR software (v19.1) measures and records the change in sensor impedance. The TDR measuring device creates a 24.4 kHz rectangle function signal with 0.5 V amplitude and a rise time (10% to 90%) of 80 ps. In order to reduce noise, the signal is averaged over 256 samples for each impedance measurement. The experimental results are compared with the 2D simulation results and 3D simulation results from chapter 3.

## 5. Results and Discussion

**Figure 9:**
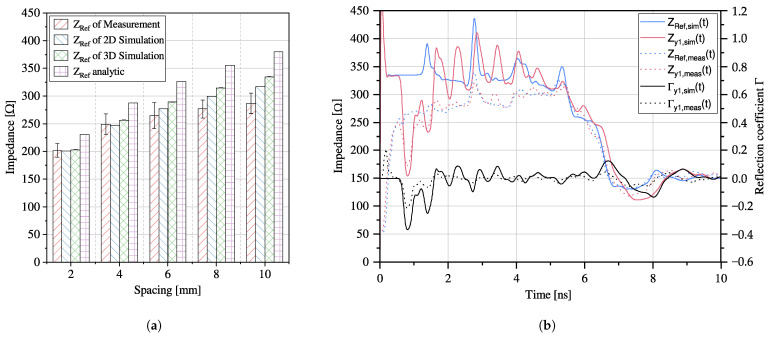
(**a**) Comparison between measurement, simulation and analytical results of the reference impedance of the transmission lines. (**b**) Measurement and simulation results of an exemplary sensor configuration s10 showing the reference impedance and the impedance with the coin on position y1 and distance 0.5 mm, as well as the resulting reflection coefficients.

A comparison between measurement and simulation results of the reference impedance of transmission lines is presented in Figure 9a. The reference impedance $Z_{\mathrm{Ref}}$ is the impedance of the sensor without the coin and PTFE spacer measured. The results of the 2D and 3D simulations are obtained using the calculation method and model presented in Section 3.2 and Section 3.3, respectively. It can be observed that the reference impedance of the sensor increases with an increase in spacing between the two traces. In general, the results obtained by the three methods are relatively consistent, especially at small spacings, where the results are almost identical. From the consistency of the results, we conclude that the simulation models are valid.

In this paper, the reflection coefficient Γ determined from the reference impedance signal, designated as $Z_{\mathrm{Ref}}\left(t\right)$, and an impedance signal with the object positioned at a specific *y*-coordinate, designated as $Z_{y}\left(t\right)$, is used to quantify the sensitivity, according to Equation (12). Both signals are time-dependent and obtained either by simulation or measurement.

(12) $$\begin{array}{c} \Gamma_{y}\left(t\right)=\frac{Z_{y}\left(t\right)-Z_{\mathrm{Ref}}\left(t\right)}{Z_{y}\left(t\right)+Z_{\mathrm{Ref}}\left(t\right)} \end{array}$$

Figure 9b shows a measurement of a sensor configuration s10 with a coin at position y1 and distance 0.5mm, where the blue lines are the reference impedances and the red lines are the impedances with the coin. The black lines are the reflection coefficients according to Equation (12). Dotted lines refer to the measurement results, whereas solid lines refer to simulation data.

The impedance signals obtained from the measurement exhibit two consecutive rising slopes, where the first slope rises rapidly from 50 Ω coaxial cable impedance to 260 Ω, followed by a slower rising slope to 320 Ω and then a falling slope ending at 150 Ω. Due to the complex signal shape, it is difficult to determine the effective impedance of the TL directly. To address this, for Figure 9a, the mean value of the slope between 260 Ω and 320 Ω is taken as the effective impedance. The standard deviation is depicted as an error bar, representing the uncertainty associated with determining the effective impedance. The signals are additionally superimposed with impedance variations of higher frequency and small amplitude. However, the impedance changes at coin position y1, which correspond to a time of 0.8 ns, vary significantly compared to the reference measurement. The maximum absolute reflection coefficient observed in this measurement is 0.2.

On the other hand, the simulated impedance signals experience a fast overshoot during the rising transition from 50 Ω to 330 Ω, exceeding the measured impedance by 30%. The signals are also superimposed with impedance fluctuations of a similar phase and frequency as the measurement, but with higher amplitudes. The falling slopes and final impedances in the simulation are comparable to the measurement data. The simulation result yields an absolute maximum reflection coefficient of 0.38.

### 5.1. Influence of Position and Spacing

**Figure 10:**
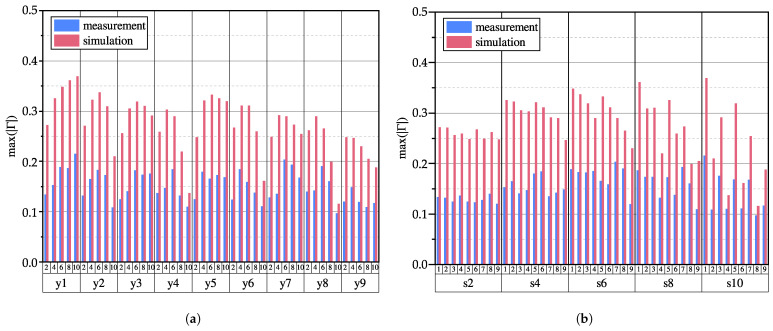
Maximum absolute reflection coefficients resulting from nine different coin positions for 5 different sensor configurations: (**a**) grouped by coin positions; (**b**) grouped by spacing.

Analogously, the impedance changes caused by the coin at nine different positions (y1 to y9) are investigated. The reflection coefficient of the coin at position 1 with a varying thickness (0.5 mm, 1 mm, 2 mm) of the PTFE spacer can be obtained similarly. Figure 10a shows the maximum absolute reflection coefficients of the coin at nine positions with a 0.5 mm PTFE spacer. From both the simulation and measurement results, it can be seen that the reflection coefficient is highest at position y1, which means that the coin is easiest to detect at position y1. In contrast, the reflection coefficient is smallest at position y9, which means that the coin is most difficult to detect at this position. This phenomenon may be attributed to interference due to reflections at the end of the transmission line, as the termination resistance is not matched to the reference impedance.

Notably, in contrast to the simulations, the measurements did not show a decrease in the reflection coefficient proportional to an increase in the coin position, which is directly related to the sensor length for all sensor configurations. Furthermore, the reflection coefficient observed in the simulations was markedly higher than that observed in the experimental measurements. The authors attribute this discrepancy to the idealized modeling approach employed in the simulations, which does not account for any damping effects resulting from power dissipation mechanisms. However, when comparing the influence of the transmission line spacing in the simulation and experiment at each position, the qualitative characteristics were well captured.

In Figure 10b, the sorting of spacing and position is reversed with respect to Figure 10a. It can be seen from the results that the difference in the reflection coefficient between the odd positions y1 to y9 and the even positions y2 to y8 is more pronounced with increased spacing. Specifically, the reflection coefficients between the positions are nearly identical for the 2 mm spacing, whereas the reflection coefficients between odd and even positions are considerably different for the 10 mm spacing. This is related to the size of the coin and the spacing of the TL. For a smaller spacing, the coin will always cover at least one TL fully independently from an even or odd position, so there is little difference in the reflection coefficient of each position. In contrast, for a larger spacing, the coin will not cover the transmission line completely in odd positions, resulting in “good” and “bad” detection positions.

**Figure 11:**
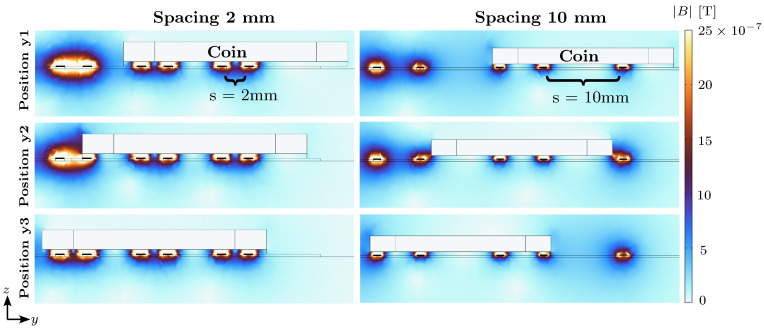
Cross-sectional view of the magnetic flux density norm around the coin and the transmission line of s2 and s10 at position y1, y2 and y3. As the coin is implemented as a surface model only, there is no magnetic field simulated inside the coin.

Figure 11 illustrates the influence of a coin on the magnetic flux density for different combinations of spacing and position. As a metallic object, the coin interacts with the magnetic field component of the electromagnetic wave propagating through the transmission line. The interaction occurs due to eddy currents induced within the conductive coin, which generates an opposing magnetic field, leading to a perturbation in the source magnetic field; hence, the coin shields the source field. Consequently, this changes the local inductance of the TL and therefore the local impedance. For any configuration, the magnitude of the inductance change is related to the degree of the local coverage of the transmission line.

Similarly, the electric field component of the electromagnetic field also interacts with the coin. When the coin covers the transmission line, most of the electric field lines will propagate through the electrically conductive coin rather than through the air, resulting in a change in the effective capacitance locally. This change in capacitance contributes to the overall change in impedance.

### 5.2. Influence of Object Distance

**Figure 12:**
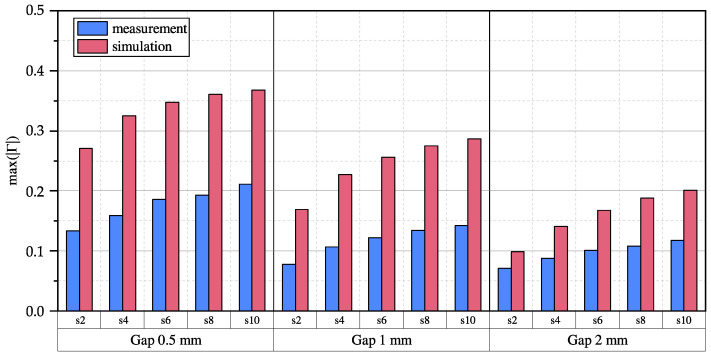
The maximum absolute reflection coefficients for different object distances from 0.5 mm to 2 mm measured for different spacings and at position y1.

Figure 12 shows the reflection coefficient for different gaps between the coin and transmission line realized with a PTFE spacer. It can be observed that the reflection coefficient becomes larger as the spacing increases for a constant gap, which was already shown and discussed in Figure 10. However, it can also be clearly observed that any further increase in spacing leads to a smaller increase in the reflection coefficient.

Furthermore, it is noticeable that the reflection coefficient increases as the gap between the coin and the transmission line decreases. This is primarily due to the fact that the closer the coin is to the transmission line, the greater the number of magnetic field lines that are deflected by the coin, and the more electric field lines passing through the coin. Therefore, the coin becomes easier to detect.

### 5.3. Summary and Limitations

In summary, the measurement results for the coin object indicate that increasing the spacing between the copper tracks of the TL leads to an increase in the maximum absolute reflection coefficient. This suggests that the coin becomes more easily detectable, partially compensating for an increased object distance. However, this effect diminishes as the spacing continues to increase, and eventually, a reversal is expected, resulting in a decrease in the reflection coefficient when the spacing becomes larger than the object itself. Thus, to effectively detect a variety of metallic objects, a careful balance between expected object sizes, distances and transmission line spacings is necessary. Figure 12 shows a rapid decline in the reflection coefficient with an increasing gap. This indicates that objects that are not located in the direct vicinity of the sensor are not detectable. This includes objects that protrude into the air gap between the sending and receiving coil but also metallic parts of the receiver itself. Additionally, based on the observed results, it is unclear whether the effect on the maximum absolute reflection coefficient is predominantly influenced by the electric or magnetic field component. Further investigations are required to gain a better understanding of this aspect.

For practical sensor applications in the real world, it is essential to have a method that effectively filters out the inductive effects of strong external magnetic fields. Fortunately, the nominal operating frequency of the WPTS is 85 kHz [1], while the TDR method operates in the range of megahertz to gigahertz frequencies. This frequency mismatch provides opportunities for the design and implementation of effective high-pass filters.

## 6. Conclusions

In this study, the influence of varying transmission line parameters on the performance of a novel TDR-based FOD detection system for wireless power transfer applications was investigated on an analytical, numerical and experimental basis. For this purpose, several laboratory-scaled prototypes based on coplanar strips with varying trace spacing were manufactured and tested for different object positions. The measurement results were compared with those obtained from 2D and 3D simulations. Although notable quantitative differences in the simulated and measured impedances were observed, the qualitative comparison of the reflection coefficients shows a very good consistency, indicating that the electromagnetic interaction mechanism is captured well in simulation. In detail, the investigation of both the magnetic flux density and the electric field strength around the coin and the transmission line revealed that the coin interacts with both the magnetic and electric field components of the electromagnetic wave, leading to changes in local inductance and capacitance, respectively. This interaction leads to a distinctive relationship between the spacing of the transmission line’s copper tracks and the size and distance of the foreign object, which has been presented in this study.

In conclusion, understanding the impact of varying transmission line parameters is crucial for optimizing FOD detection systems based on time domain reflectometry. As the general detection principle has been proven to work for various distances and without blind spots, this study provides valuable insights into the design considerations of the underlying transmission lines and offers a wide basis for future research in FOD detection system performance. However, a full comparison to conventional FOD systems is currently not feasible, as critical aspects such as electromagnetic compatibility and scalability have yet to be determined. Thus, in the future, the authors will focus on sensor scalability, the extension of the foreign object catalog to comply with the recommended objects in SAE J2954 [1] and on methods for the suppression of electromagnetic interference.

## Figures and Tables

**Table 1:** Dimensional data of sensors for simulation.

Designation	Description	Unit	Range of Values
*s*	distance between the two traces	mm	2; 4; 6; 8; 10
$h_{\mathrm{spacer}}$	gap between coin and transmission line	mm	0.5; 1; 2

**Table 2:** Parameter data for the simulation.

Material	Relative Permittivity $\epsilon_{r}$ [1]	Relative Permeability $\mu_{r}$ [1]	Electrical Conductivity σ [S/m]	Reference
Air	1	1	0	-
PTFE	2.1	1	0	[33]
RO4350B	3.66	1	0	[32]

**Table 3:** Data of the geometry with different parameters.

Designation	$d_{1}$ [mm]	$d_{2}$ [mm]	$l_{\mathrm{subs}}$ [mm]	$w_{\mathrm{subs}}$ [mm]
s2	5	7.5	160	55
s4	5	6.5	160	65
s6	4.5	6	160	75
s8	4.5	6	160	85
s10	4.5	6	160	95

## Data Availability

Data are contained within the article.

## Abbreviations

The following abbreviations are used in this manuscript:

FEM	finite element method
FOD	foreign object detection
GA	ground assembly
LOD	living object detection
MOD	metal object detection
PCB	printed circuit board
PEC	perfect electric conductor
PTFE	polytetrafluoroethylene
SBC	scattering boundary condition
TDR	time domain reflectometry
TL	transmission line
WPT	wireless power transfer
WPTS	wireless power transfer system
